# Which positive factors give general practitioners job satisfaction and make general practice a rewarding career? A European multicentric qualitative research by the European general practice research network

**DOI:** 10.1186/s12875-019-0985-9

**Published:** 2019-08-09

**Authors:** B. Le Floch, H. Bastiaens, J. Y. Le Reste, H. Lingner, R. Hoffman, S. Czachowski, R. Assenova, T. H. Koskela, Z. Klemenc-Ketis, P. Nabbe, A. Sowinska, T. Montier, L. Peremans

**Affiliations:** 1 EA 7479 SPURBO, Department of General Practice, Université de Bretagne Occidentale, Brest, France; 2 Department of Primary and Interdisciplinary Care. Faculty of Medicine and Health Sciences, University Antwerp, Antwerp, Belgium; 3 Centre for Public Health and Healthcare, Hannover Medical School, Hannover, Germany; 4 Department of Family Medicine, Tel Aviv University, Tel Aviv, Israel; 5 Clinical Psychology Department, Nicolaus Copernicus University, Torun, Poland; 6 Department of Urology and General Medicine, Department of General Medicine, Faculty of Medicine, Medical University of Plovdiv, Plovdiv, Bulgaria; 7 University of Tampere, Faculty of Medicine and Life Sciences, Tampere, Finland; 8 Department of Family Medicine, Faculty of Medicine, University of Ljubljana, Ljubljana, Slovenia; 9 Department of Family Medicine, Faculty of Medicine, University of Maribor, Maribor, Slovenia; 10 Facultad de Humanidades, Universidad Católica del Norte, Antofagasta, Chile; 11 Escuela de Inglés, Casa Central, Angamos, 0610 Antofagasta, Chile; 12 Department of English, Nicolaus Copernicus University, Torun, Poland; 13 Unité INSERM 1078, SFR 148 ScInBioS, Université Européenne de Bretagne, Faculté de Médecine et des Sciences de la Santé, Brest, France; 14 Department of Nursing and Midwifery. Faculty of Medicine and Health Sciences, University Antwerp, Antwerp, Belgium; 15 Mental Health and Wellbeing Research Group, Vrije Universiteit Brussel, Brussel, Belgium

**Keywords:** Adult, Career choice, Career mobility, Family practice, General practitioners, Health care system, Humans, Job satisfaction, Physician, Primary health care

## Abstract

**Background:**

General Practice (GP) seems to be perceived as less attractive throughout Europe. Most of the policies on the subject focused on negative factors. An EGPRN research team from eight participating countries was created in order to clarify the positive factors involved in appeals and retention in GP throughout Europe. The objective was to explore the positive factors supporting the satisfaction of General Practitioners (GPs) in clinical practice throughout Europe.

**Method:**

Qualitative study, employing face-to-face interviews and focus groups using a phenomenological approach. The setting was primary care in eight European countries: France, Belgium, Germany, Slovenia, Bulgaria, Finland, Poland and Israel. A thematic qualitative analysis was performed following the process described by Braun and Clarke. Codebooks were generated in each country. After translation and back translation of these codebooks, the team clarified and compared the codes and constructed one international codebook used for further coding.

**Results:**

A purposive sample of 183 GPs, providing primary care to patients in their daily clinical practice, was interviewed across eight countries. The international codebook included 31 interpretative codes and six themes. Five positive themes were common among all the countries involved across Europe: the GP as a person, special skills needed in practice, doctor-patient relationship, freedom in the practice and supportive factors for work-life balance. One theme was not found in Poland or Slovenia: teaching and learning.

**Conclusion:**

This study identified positive factors which give GPs job satisfaction in their clinical practice. This description focused on the human needs of a GP. They need to have freedom to choose their working environment and to organize their practice to suit themselves. In addition, they need to have access to professional education so they can develop specific skills for General Practice, and also strengthen doctor-patient relationships. Stakeholders should consider these factors when seeking to increase the GP workforce.

## Background

The low appeal of General Practice and primary care as a career option is a recurrent problem for healthcare systems throughout Europe, USA and other countries in the Organization for Economic Cooperation and Development (OECD) [1, 2]. A high-performing primary healthcare workforce is necessary for an effective health system. However the shortage of health personnel, the inefficient deployment of those available, and an inadequate working environment contribute to shortages of consistent and efficient human resources for health in European countries.

The European Commission projects the shortage of health personnel in the European Union to be 2 million, including 230,000 physicians and 600,000 nurses, by the year 2020, if nothing is done to adjust measures for recruitment and retention of the workforce [3]. Research has shown a strong workforce in General Practice is needed to achieve an efficiency balance between the use of economic resources and efficient care for patients. [4].

Most of the research focused on the GP workforce concentrated on negative factors. The reasons students did not choose this as a career or GPs were leaving the profession were widely explored. Burnout was one of the most frequently highlighted factors [5]. In many OECD countries, apart from the United Kingdom, the income gap between GPs and specialists had expanded during the last decade, promoting the appeal of other specialties for future physicians [6]. Health policy makers, aware of the problem of a decreasing General Practice workforce, tried to change national policies in most European countries to strengthen General Practice. Health professionals respond to incentives but financial incentives alone are not enough to improve retention and recruitment. Policy responses need to be multifaceted [7]. Dissatisfaction was associated with heavy workload, high-levels of mental strain, managing complex care, expectations of patients, administrative tasks and work-home conflicts. Focusing on these issues created a negative atmosphere [5, 8–10]. In the above mentioned report of the European commission on recruitment and retention of Workforce in Europe, the authors used a model of Huicho et al. as a conceptual framework to analyze the situation [11]. Attractiveness and retention are two outputs used in the model. Retention is determined by job satisfaction and duration in the profession.

The concept of job satisfaction is complex as it changes over time according to social context. “Job satisfaction is a pleasant or positive emotional state resulting from an individual’s assessment of his or her work or work experience” [12]. There is a weak relationship between enjoyment and satisfaction, suggesting that other factors contribute to job satisfaction [13, 14]. Furthermore, general practice is a specific field and theories on job satisfaction in this field are not fully explained by theories on human motivation in general. According to the research group hypothesis, it was important to investigate the positive angle separately in order to understand which factors give GPs job satisfaction. That was the focus chosen by the research team.

The literature highlighted the poor quality of the research about job satisfaction within European General Practice. Most studies were carried out by questionnaire [15], focusing on issues of health organization or business and did not reach the core of GP daily practice. Some studies had confusion bias caused by authors’ pre-requisites on the attractiveness of General Practice [16]. Surprisingly few qualitative studies explored the topic of satisfaction [17, 18]. Literature did not show an overall view of GPs’ perception of their profession. It was not certain that these positive factors were similar across different cultures or in different healthcare contexts. Consequently, research into positive factors, which could retain GPs in practice, would help to provide a deeper insight into these phenomena.

The aim was to explore the positive factors supporting the satisfaction of General Practitioners (GPs) in primary care throughout Europe.

## Method

This research is descriptive qualitative study on positive factors for attractiveness and retention of General Practitioners in Europe.

### Research network

A step-by-step methodology was adopted. The first step was to create a group for collaborative research [19, 20]. The EGPRN created a research group involving researchers from any country wishing to participate: Belgium (University of Antwerp), France (University of Brest), Germany (University of Hannover) and Israel (University of Tel Aviv), Poland (Nicolaus Copernicus University), Bulgaria (University of Plovdiv), Finland (University of Tampere) and Slovenia (University of Ljubljana). Undertaking such a study in several different countries, with different cultures and different healthcare systems, presented a challenge. This has been made possible by the support of the EGPRN in the various meetings held throughout Europe.

**Fig. 1:**
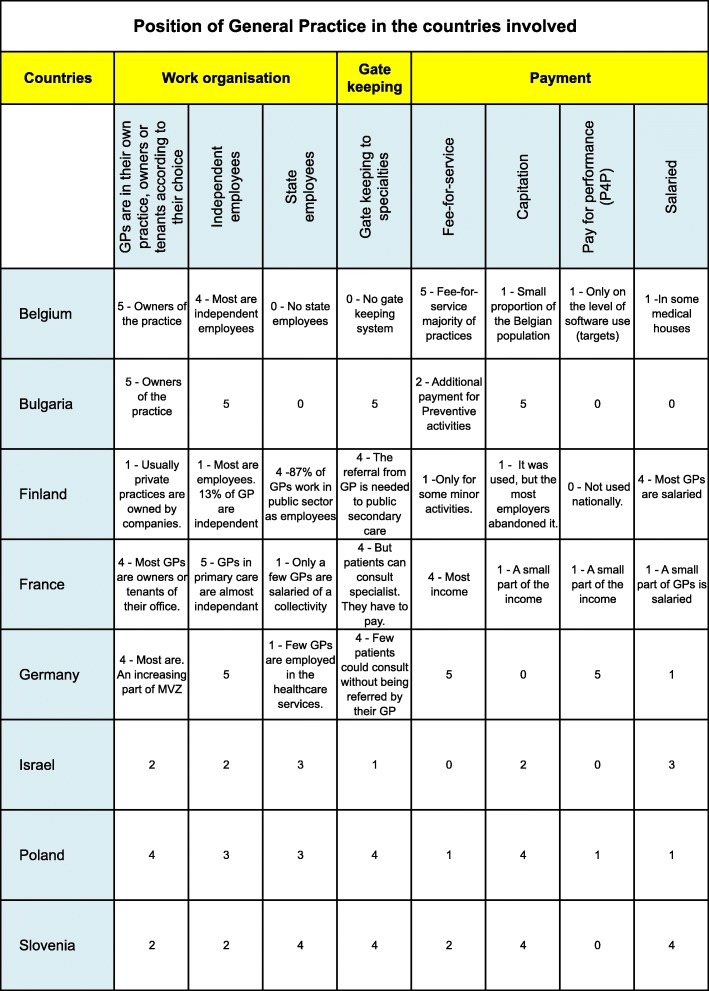
Position of General Practice in the countries involved

Figure 1 gives an overview of the position of the general practitioner in each country, according to the different healthcare systems.

The authors scored the importance of some specificities of practice in their own country from 0 (not important) to 5 (very important).

The research team decided to conduct a descriptive qualitative research study, from GPs’ perspective, in each participating country [21, 22]. The first interviews were completed in the Faculty of Brest, in France. The aim was to pilot the first in-depth topic guide.

### Participants

GPs were purposively selected locally using snowballing in each country. Participants were registered GPs working in primary care settings. To ensure diversity, the following variables were used: age, gender, practice characteristics (individual or group practices), payment system (fee for service, salaried), teaching or having additional professional activities. The GPs included provided their written informed consent. GPs were included until data saturation was reached in each country (meaning no new themes emerged from the interviews) [21, 23, 24].

Overall, 183 GPs were interviewed in eight different countries: 7 in Belgium, 14 in Bulgaria, 30 in Finland, 71 in France, 22 in Germany, 19 in Israel, 14 in Poland and 6 in Slovenia. In each country, the principle of obtaining a purposive sample was observed and GPs were recruited until data sufficiency was reached. Four qualitative studies were achieved in France. In France, it was always the intention to include more participants than in the other countries, with a view to exploring potential differences between practice locality, gender, type of practice and teaching activities. One study was carried out by five focus groups, which brought together 38 GPs; the three other studies used individual interviews (11 participants, 6 participants, and 14 participants). The other countries conducted one qualitative study each. The research activities were undertaken in Germany by focus groups, in Israel using focus groups and individual interviews and in the other countries by individual interviews.

### Study procedure and data collection

The research team discussed every step of the study, in two annual workshops, during EGPRN conferences, within the duration of the study.

As there were few examples in the literature and, as the existing models of job satisfaction were more oriented towards employees working in a company, the international research team developed an interview guide based on their previous literature review [16]. The guide was piloted in France and was adapted and translated to ensure a detailed contribution from the GPs interviewed and, subsequently, a rich collection of qualitative data in each country. Local researchers conducted the interviews in their native language. In accordance with the research question interviewers were looking for positive views. Overall interviewers were GPs working in clinical practice and in a university of college, except in Belgium where the interviewer was a female psychologist, working in the department of GP. The GPs were first asked to give a brief account of a positive experience in their practice (ice-breaker question) [21]. The interview guide (Table 1) was used to encourage participants to tell their personal stories, not to generate general ideas but to focus on positive aspects.

**Table 1:** interview guide

Topic 1	In the life of a General Practitioner, there are pleasant experiences. Could you tell us about one?
Topic 2	What makes you happy in the profession of General Practitioner? What motivates you to go to work every morning? Factors related to the job content
Topic 3	Factors concerning a satisfying practice organization, location, collaboration
Topic 4	What makes for work-life balance, especially where the family is concerned?
Topic 5	The significance of the GP’s residential environment?
Topic 6	Coping strategies to overcome difficulties

To ensure a maximal variation in collection techniques, in order to collect both individual and group points of views, interviews and focus groups had to take place. Saturation (no new themes emerging from data) had to be reached in each country [21].

### Data analysis

A thematic qualitative analysis was performed following the process described by Braun and Clarke [25].

In each country, at least two researchers inductively and independently analyzed the transcripts in their native language using descriptive and interpretative codes. They issued a verbatim transcript of one particular part of, or sentence from, the interview to illustrate every code in the codebook. Each code was extracted in the native language and translated into English. The contextual factors were explored in each setting by the local team of researchers and these factors were taken into account during the analysis. Then the whole team discussed the codes several times in face-to-face meetings during seven EGPRN workshop meetings. The research team merged the national codes into one unique European codebook. During a two-day meeting, the research team performed an in-depth exploration of interpretative codes and a final list of major themes was generated. Credibility was verified by researcher triangulation, especially during data collection and analysis. During the EGPRN workshops, peer debriefings on the analysis and the emerging results were held. Interviewers and researchers from such diverse backgrounds as psychology, sociology, medicine and anthropology reflected on the data from their own researcher’s perspective.

## Results

Table 2 gives an overview of the characteristics of the participants. The mean age was high which is an indication of a long duration in the profession.

**Table 2:** Characteristics of the GPs interviewed

Characteristics of the GPs interviewed											
Country	Number of GPs interviewed	Gender		Age average	Type of Practice		Practice Location			Teaching	
		M	F		Single-handed	Group	Urban	Semi-rural	Rural	Yes	No
Belgium	7	4	3	52	3	4	4	3	0	4	3
Bulgaria	14	3	11	50	11	3	11	2	1	n/a	n/a
Finland	30	14	16	49	10	30	17	3	10	6	24
France	71	35	36	49	25	46	40	5	26	28	43
Germany	22	13	9	52	12	10	8	0	14	11	11
Israel	19	6	13	51	n/a	n/a	17	0	2	n/a	n/a
Poland	14	5	9	47	8	6	8	2	4	5	9
Slovenia	6	2	4	55	4	2	0	3	3	2	4
Total	183	82	101	50	74	100	106	20	57	56	93

**Fig. 2:**
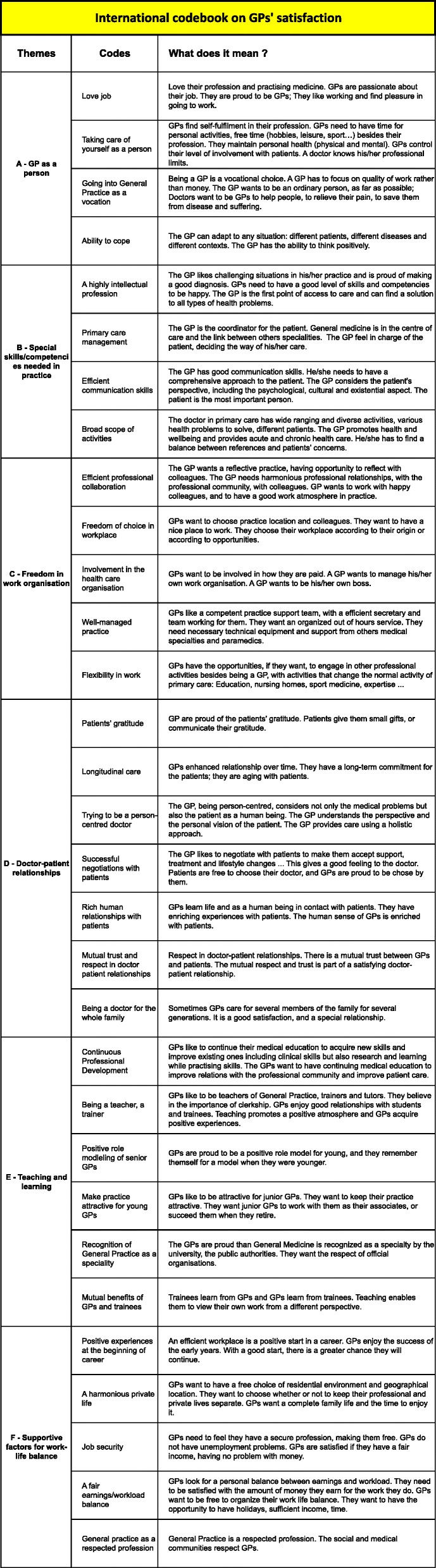
International codebook on GP satisfaction

Six main themes were found during analysis. The results are summarized in the Fig. 2: International codebook on GP satisfaction.

### GP as a person

The analysis of the data showed that the GP was a person with intrinsic characteristics, including interest in people’s lives, with a strong ability to cope with different situations and patients. GPs loved to practice and the passion for their job was more important than the financial implications.

*“I also work with a very heterogeneous population, ultra-religious and secular, from various countries of origin” (Israel).*

*“Really pleasant to work with patients, it’s not only the financial aspect” (Bulgaria).*

*“I work for pleasure. I don’t do it for the money. If I don’t like it anymore I’ll stop doing it” (Belgium)*

GPs said they wanted to stay ordinary people with a strong need to take care of their personal wellbeing. This was more than just having time for hobbies and leisure. GPs were looking for other intellectual challenges and personally enriching activities in their free time.

*“General practice is a beautiful profession but you are on your own too much, even in a group practice. You see the community from a limited perspective. It’s important to keep in touch with the community. The fact remains that you are probably a father or mother or a partner, as well as being a physician. It’s interesting to have a different perspective: it broadens your way of thinking. Reading books is the same. It’s essential to read good books and to empathize with the characters. This is enriching for you as a human being, but also for your practice.” (Belgium).*

GPs said they wanted to be there for their patients, to find common ground with them, but they also wanted to control the level of involvement with their patients. They described the ability to balance empathy with professional distance in their interaction with patients and being able to deal with uncertainty in the profession.

The GP as a person theme was important, as all the above conditions were required in order to be a satisfied GP who wishes to remain in clinical work

### GP skills and competencies needed in practice

GPs reported satisfaction about making correct diagnoses in challenging situations, with low technical support, and being rewarded with patients’ gratitude. The intellectual aspect of medical decision-making led to effective medical management and was a positive factor for GPs. General practice is the first point of care for the patient and GPs felt themselves to be the coordinators and managers of care and the advocates for the patient. To be the key person in primary care requires strong inter-professional, collaborative skills and effective support from other medical specialties and from paramedics.

GPs believed that it was highly important to be an efficient communicator to perform all these tasks. GPs were patient-centered and wanted to provide care using a comprehensive and a holistic approach. A patient centered approach is a WONCA core competency of General Practice while efficient communication with the patient is a generic skill for all health workers.

They wanted to bring together a broad medical knowledge with a high level of empathy, balancing the patient’s concerns with official guidelines. Guiding the patient’s education was an important role for the GP, who was also a coach for life style changes. This theme was linked to the holistic model for General Practice which is also a WONCA’s core competency.

*“To be both competent and do a bit of everything” (France).*

*“This is intellectually extremely stimulating and challenging work” (Finland).*

*“Happy and satisfied when making the correct diagnoses” (Bulgaria).*

*“The patient arrives and thanks me for the good diagnoses” (Poland).*

*“You don’t just see common colds during the day. You get interesting cases and you have time to explore them. This makes general practice interesting. It’s a 360° job. Variation is important”. “It’s our task to empower young Muslims to encourage them to study well, to become nurses or physicians”. Belgian GP*

### Doctor-patient relationships

Patients are free to choose their GP and this is important because of the particular aspects of the doctor-patient relationship in primary care. There was a strong relationship between the GP as a person and the GP who enjoyed a rewarding, interpersonal relationship with patients. GPs had enriching human experiences with patients which was important to the physician’s self-fulfillment as a human being. Mutual trust and respect in their relationships were important dimensions. Being a patient-centered physician was a rewarding challenge.

GPs felt they were a part of the patient’s environment, but with the need to set their professional limits. GPs learned about life through their patients.

GPs said they were ageing with their patients and had a long-term relationship with some of them. They were “real family doctors” and often cared for several generations.

They saw babies grow up and become parents themselves. These unique doctor-patient relationships enhanced GP satisfaction.

*“I am the doctor for this whole family and in general practice that is something important” (France).*

*“Some I got to know when they were small kids and they still come to see me at the age of 18 or older.” (Germany).*

*“We know much more about them than other doctors, because our patients have chosen us” (Bulgaria).*

*“We accompany patients, throughout pregnancy, cancer and death and from the moment before birth until the age of 99 years and over” (Germany).*

*“Patients asked for a home visit and insisted I join them at their meal and sometimes I did that but only when they were more like friends… I’ve had a lot of invitations to weddings…” (Belgium)*

GPs also liked to negotiate with patients, to help them to make decisions but also to motivate them to make lifestyle changes.

### Autonomy in the workplace

Freedom in practice was closely related to work organization, which was important in all countries.

GPs stayed in clinical work if they had chosen their own practice location. The living environment needed to be attractive for the family. GPs wanted to apply personal touches to their consulting rooms, to make choices in the technical equipment they used which suited their personal requirements.

Even more important was the possibility of choosing work colleagues who shared the same vision of General Practice. Satisfied GPs contributed to the organization of the practice and were influential in decisions about work and payment methods. Where there was a salaried system, GPs wanted to earn a reasonable salary to have a satisfying work-life balance.

Flexibility at work was not to be interpreted as a demand from the management to be flexible in working hours but to have the flexibility to make one’s own choices. Most GPs preferred additional career opportunities such as teaching, working in a nursing home and conducting research. To fulfil all these conditions GPs wanted to work in a well-organized practice with a competent support team, with a secretarial service, practice assistants and the necessary technical equipment.

Another condition was an organized out-of-hours service. GPs did not want to be disturbed outside practice hours without prior arrangement.

*“This is the most important in our practice that I decide when and how to work” (Bulgaria).*

*“If someone says that a practice room must be completely impersonal, it has to be interchangeable. I understand this. It’s respectful towards the others but a personal touch is important for communicating something about yourself to the patient. That is important.” (Israel).*

*“It is important to have one’s own organizational systems and equipment” (France).*

*“I didn’t have to do night shifts” (Poland).*

### Teaching general practice

GPs reported that they wanted to acquire new medical knowledge and learn new techniques. They liked to transmit the skills of their job. They were proud of their profession and they wanted to teach and to have an effective relationship with trainees. Teaching contributed to feelings of satisfaction with the profession. GPs mentioned the importance of training in attracting junior colleagues and the positive aspect of the mutual benefit to GPs and trainees. Teaching gave GPs more incentives for their own continued professional development and enabled them to complete their competencies. GPs feel gratified where general medicine is recognized as a specialty at the university and by the public authorities.

*“Guiding younger colleagues is the most rewarding part of my job” (Finland).*

*“I like to transmit what I have learned” (France).*

*“I was a tutor for a seminar group, teaching, I like to do that, those people had to learn, that was very pleasant” (Belgium).*

*“I am teaching General Practice to students and I have found I have a flair for it. It is really fun!” (Germany).*

*“I feel good accompanying young trainees through the process of making their choices” (Belgium).*

*“All that you do in teaching (trainees), transmitting your knowledge to another, improves your accumulated experience. You see yourself through the eyes of others” (Israel).*

### Supportive factors for work-life balance

Factors that supported an efficient work-life balance were the possibility of having a full family life, with a social support network and the opportunity to benefit the whole family by enjoying holidays, money and free time. Money was not the most important issue, but income needed to be sufficient for a comfortable family life, meaning sufficient resources for a satisfying education for the children and the possibility of having regular holidays. GPs found they have job security which enables them to feel secure and free from unemployment worries.

GPs explained that they wanted to choose how to separate professional and private life. They said they wanted to have social contacts in the community, which would give them a broader perspective in terms of their patients. Having relationships with patients outside the practice was important. GPs said they needed to be part of the social community if they were to stay in General Practice. GPs wanted to have a full family life and to keep free time for this.

*“I could have my family, my wife involved” (Poland).*

*“I try to keep my Wednesday afternoon free to stay at home. I now set out my priorities. If something happens with the children, I change my work” (Belgium).*

*“Family Medicine is an opportunity to be with the family” (Israel).*

*“My family supports me” (Bulgaria).*

*“I try to keep work and leisure time away from each other... It is important in terms of coping. In my leisure time I have a different role from that of a doctor” (Finland)*

### Country specific themes

Besides those international themes there were some country specific results.

In Poland and in Slovenia even when they were prompted in the interviews, GPs did not mention the importance of teaching.

Belgian GPs said how important discussing the vision and mission involved in starting a group practice was to them. They took time for this process and wanted junior colleagues in practice who would share their vision and their mission. Statements needed to be updated regularly to meet the needs of a changing society and the challenges in health care. Group practices used external coaching to overcome problems.

*Vision and mission are important. We started from ten values as respect, diversity, the aim to train young GPs…. You have to renew the vision and mission regularly and to adapt at the changing community. Belgian male GP*

French GPs were very attentive to the need for organized continuity of care. The GPs wanted to be there for their patients, but they also wanted to protect their personal lives. The word “vocation” had a religious connotation that displeased some GPs.

Finish GPs appreciated the stimulating working community and multidisciplinary teamwork. In addition, they valued the set working hours and professional development work available in the workplace.

Israeli GPs were proud of their respected position. They preferred a private practice in their own style and stressed the importance of teamwork.

*The clinics were, I felt good were clinics that the staff was amazing and enlisted, the nurses were good and the secretaries did the work and there was a feeling that we were working for better medicine. There were weekly meetings where we really thought how to do better, a feeling of teamwork.*

For Polish GPs, there were some positive developments in financing medicine, which were providing better opportunities for an effective work-life balance. In Poland, there was a theme, which favoured having a strong union that can influence policy. It gave the GPs an identity as a group.

*The fact that I work here as I work, my income is not too high, but still is, make it possible that my kids can attend private schools and don’t have to go to normal state schools. Polish female GP*

## Discussion

### Main results

Throughout Europe, common positive factors were found for satisfaction of GPs in clinical practice. One of the main characteristics of GPs was the need for specific competencies for managing care and communicating with patients. They needed to cope with problems during their career and professional collaboration. GPs were stimulated by intellectual challenges, not only within the profession but they also wanted enough time for personal development outside the workplace, to counterbalance the stress of daily practice.

Positive GPs are persons with intrinsic specific characteristics (open-minded, curious). Participants described themselves as feeling comfortable in their job when they were trained in specific clinical and technical skill areas and had efficient communication skills. The long-term doctor-patient relationship is perceived positively by the GPs. They love teaching all these specific skills to younger GPs and appreciate the feedback and mutual benefit to be found in teaching activities. Finally, GPs need policy support for well-managed practices and out-of-hours services to maintain their optimal work-life balance.

### Strengths and limitations of this study

To our knowledge, this multinational data analysis from 183 GPs is the first European multicentre qualitative study on this topic [16, 26]. This study collected complete and complex data from eight countries. One of the strengths was to study a diverse population of GPs, with different cultures and health systems. Despite these differences, the main satisfaction factors to become a GP and to stay in clinical practice are found in all contexts. For instance, money is important, but it’s relative because the idea to have enough to lead a comfortable family life with enough free time is for every GP crucial, although income might vary over Europe.

#### Credibility and transferability

Credibility was verified by researcher triangulation, especially during data collection and analysis. During the workshops, peer debriefings on the analysis and the emerging results were held. Interviewers and researchers from such diverse backgrounds as psychology, sociology, medicine and anthropology reflected on the data from their own researcher’s perspective. As the results in several countries with different healthcare systems were very similar, the transferability of data seems possible.

The main weakness was a possible interpretation bias. The 183 GPs provided very rich data in several languages. It was the strength of this research, but also a difficulty. The analysis and interpretation of the verbatim analysis was a linguistic and cultural problem. A different classification of themes could be achieved, but this was limited by the group meetings and the massive number of emails, phone discussions and Skype® discussions required during the research process.

The number of GPs interviewed varied in the different countries, potentially leading to differences in the informational detail and in the depth of the analysis of the interviews/focus groups. However, data saturation was reached in all settings, limiting this possible bias.

### Discussion of the findings

The theme “GP as a person” was highlighted in this study and in the literature review [16]. The studies found this special identity for GPs was linked to their intrinsic characteristics. The theme of “GP as a person” was important in each of the European countries. A GP is, of necessity, someone with a specific personality, which is suited to General Practice. GPs like to take care of people [27] *Feeling of caring »* [28]. “*I can have a big impact on people’s lives*” [27]. This is a strong personality characteristic in a GP which policy-makers might take into consideration when formulating policies which concern the medical workforce.

The GP skills and competencies were found in literature [16, 29] but in a more restricted form. They focused on an effective medical management of the patient and the subsequent feeling of being competent. In a Scottish qualitative study, GPs highlighted the satisfaction derived from the perception of the consultation outcome. *“Although clinical competence was an integral part of the doctors’ satisfaction, they alluded to personal attributes that contributed to their individual identity as a doctor”* [30]. “*Take care of them and do the best you can”* [27]. In our study we identified all WONCA core competencies and this is important [4]. Validation of WONCA’s characteristics and competencies in hundreds of interviews across eight European countries shows the strength of the WONCA theorem and common characteristics between GPs wherever they work. The analysis of the data demonstrated a strong link between competence and satisfaction. It is necessary to give general practitioners the opportunity to acquire and improve these skills.

The importance of the doctor-patient relationship was described as an effective factor in job satisfaction for the General Practice workforce [31, 32]. Nevertheless, previous studies concentrated less on the rewarding nature of the relationship, its long duration and the mutual interaction.

Freedom to manage the workplace organization has been described and is confirmed here. It does not prevent long working hours but focuses on the organization of the practice [33–35]. There was consistent evidence that GPs needed freedom for work satisfaction [36]. GPs wanted autonomy in their work [17].

The teaching and learning activities have been described and this study confirmed their importance. Academic responsibilities provide positive stimulation and new perspectives for GPs [17, 36, 37]. They wanted to be recognized by the academic world. Clerkships in General Practice were seen as important for attracting students to a career in General Practice [38]. The influence on students was important for their career choice [39]. The practice of clinical teaching in initial medical education, with positive role modelling, was also important [40, 41].

There was a strong link between the GP, his/her family and the community they are living in. This was especially true for those practising in rural areas [39] [42]. The GP’s family was sensitive to the fact that General Practice is a respected profession. Outside their professional role, other forms of satisfaction were important, such as having strong social support from schools, leisure activities and a satisfying quality of life in the residential environment [43], and of course, the importance of an income in balance with their heavy workload.

Finally, the results highlighted a particular theory to describe GP satisfaction which focuses on human relationships, specific competencies, patients and the social community.

### Implications for medical education and practice

Learning the core competencies of General Practice in initial and continuous medical education is very important and should lead to extended educational programs in Europe.

Mobilizing stakeholders is a necessary condition of success however it is not sufficient [7].

To improve the attractiveness of general practice, universities should organise a specific selection process for GPs, not just for specialists. This might engender greater respect for the profession.

Roos et al. performed a study by questionnaire on the “motivation for career choice and job satisfaction of GP trainees and newly qualified GPs across Europe” [15]. The most frequently cited reasons for choosing General Practice were “compatibility with family life,” “challenging, medically broad discipline”, “individual approach to people”, “holistic approach” and “autonomy and independence”. The current study has focused on working GPs and not on trainees, but some of the results overlap Roos’ research.

It remains essential to teach undergraduate medical students the bio-medical aspects of general practice, but it is also necessary to teach the management of primary care, interprofessional collaboration and communication skills. Trainees need to think about their own wellbeing and to learn to cope with problems in daily practice. The intellectual aspect of General Practice is important. Decision-makers should use all the means at their disposal to promote the profession by providing continual development.

GPs want to be involved in the management of their practice. Stakeholders should be aware and very cautious about this topic which is described as extraordinarily sensitive. Systems that try to administrate GP practices, without involving the GPs, should be aware that they will experience difficulties.

### Implications for research

Further studies would be useful with the objective of studying which satisfaction factors have the greatest impact on recruitment and retention in General Practice.

This description of satisfied GPs will be disseminated throughout Europe to implement new policies for a stronger GP workforce. This may assist the international research team in the design of further studies to investigate the links between these positive factors and the growth of the GP workforce. At this stage, the research team will test the usefulness of each positive factor in helping each country to design efficient policies to increase its workforce.

## Conclusion

Throughout Europe, GPs experience the same positive factors which support them in their careers in clinical practice. The central idea is the GP as a person who needs continuous support and professional development of special skills which are derived from the WONCA’s core competencies. In addition, GPs want to have freedom to choose their working environment and organize their own practice and work in collaboration with other health workers and patients.

National policy arrangements on working conditions, income, training and official recognition of general practitioners are important in facilitating the choice of a career in general practice. Stakeholders should be aware of these factors when considering how to increase the GP workforce.

## Data Availability

Some data in this study are confidential. The data generated and analyzed during the current study are not publicly available. But the datasets generated analysed during the current study are available from the corresponding author on reasonable request.

## Abbreviations

EGPRN: European General Practice Research Network
GP: General practitioner
GPs: General practitioners
n/a: not applicable
UBO: Université de Bretagne Occidentale, France.
WONCA: World Organization of National Colleges, Academies and Academic Associations of General Practitioners/Family Physicians
